# Inhibition of Autophagy Enhances Curcumin United light irradiation-induced Oxidative Stress and Tumor Growth Suppression in Human Melanoma Cells

**DOI:** 10.1038/srep31383

**Published:** 2016-08-09

**Authors:** Tianhui Niu, Yan Tian, Zhusong Mei, Guangjin Guo

**Affiliations:** 1 Aviation Medicine Research Laboratory, The General Hospital of the Air Force, Beijing, China; 2 Department of Dermatology, The General Hospital of the Air Force, Beijing, China

## Abstract

Malignant melanoma is the most aggressive form of skin carcinoma, which possesses fast propagating and highly invasive characteristics. Curcumin is a natural phenol compound that has various biological activities, such as anti-proliferative and apoptosis-accelerating impacts on tumor cells. Unfortunately, the therapeutical activities of Cur are severely hindered due to its extremely low bioavailability. In this study, a cooperative therapy of low concentration Cur combined with red united blue light irradiation was performed to inspect the synergistic effects on the apoptosis, proliferation and autophagy in human melanoma A375 cell. The results showed that red united blue light irradiation efficaciously synergized with Cur to trigger oxidative stress-mediated cell death, induce apoptosis and inhibit cell proliferation. Meanwhile, Western blotting revealed that combined disposure induced the formation of autophagosomes. Conversely, inhibition of the autophagy enhanced apoptosis, obstructed cell cycle arrest and induced reversible proliferation arrest to senescence. These findings suggest that Cur combined with red united blue light irradiation could generate photochemo-preventive effects via enhancing apoptosis and triggering autophagy, and pharmacological inhibition of autophagy convert reversible arrested cells to senescence, therefore reducing the possibility that damaged cells might escape programmed death.

Melanoma is a skin neoplasm originating from melanocytes, which are specialized pigment-producing cells in the basal layer of the epidermis^1^,^2^. Malignant melanoma is the deadliest modality of skin carcinoma that possesses fast proliferation rate and highly invasive characteristics^1^,^3^. In the USA, more than 7000 persons die from malignant melanoma every year, causing a heavy burden to the society^4^. Although the basic resistance of melanoma to drugs is most likely due to the abnormal regulation of apoptosis, the therapy of melanoma remains a complex issue requiring a multidisciplinary approach^4^. So far, the combination of phototherapy and chemotherapy is considered to be an efficient method to lessen the dose of chemotherapeutic drugs and reduce the harmful side effect^5^,^6^.

Phototherapy with visible light has attracted more and more interests in dermatological treatment. Blue light, a UV-free irradiation with a wavelength range of 400–480 nm, shows low toxicity and adverse effects to mammalian cells compared with ultraviolet irradiation^7^, except when used at high concentration dosages which could cause severe diverse reactions^8^,^9^. Additionally, blue light has attracted increasing attention due to its innate anti-proliferative function without adding exogenous photo-sensitizing agents^9^. Red light, a portion of visible light ranging from 620 nm to 770 nm, has been well received in photodynamic therapy (PDT) because of its puncture capacity to profoundly penetrate the skin layer to about 6 mm^10^. Red light may possess the anti-inflammatory ability by affecting the release of cytokines from macrophages or other cells as well as the capability to restrain angiogenesis via motivating other chromophores, nevertheless, the accurate mode of action of red light is still incompletely understood^11^,^12^.

Curcumin (Cur) is a bioactive compound extracted from the rhizome of *Curcuma longa L*in., and possesses diverse pharmacologic effects, including anti-inflammatory, anti-bacterial, apoptosis-inducing and tumor growth suppressing properties^13^,^14^. Unfortunately, the extremely low biological availability of Cur, which may be resulted from poor assimilation and fast systemic elimination, significantly reduces its therapeutic advantage^15^,^16^. Most of studies showed that Cur induced apoptosis and suppression of cell proliferation were mainly at high concentrations ranging from 10 to 150 μM, in different tumor cells^6^. As a natural photochemical, Cur has a wider range of absorption peak (from 300 nm to 500 nm), and exhibits the highest absorption at about 420 nm when combined with visible light^5^. When combined with visible light irradiation, the effects of Cur were enhanced due to the increased light energy intake under these occasions^17^,^18^. Therefore, Cur may be applied as a photosensitizer widely spreading in the PDT at low concentrations. Studies have proved that Cur exerts its cytotoxic effects through regulating multiple signaling pathways^19^. For example, Cur regulates intracellular signaling pathways involving mitogen-activated protein kinases (MAP-kinases), transcription factor NF-*k* B as well as signal transducer and activator (STAT)^20^,^21^,^22^. Some studies show that Cur promotes cell cycle arrest and inhibits cell survival by negative modulation of the PI3K/AKT signaling pathway^23^,^24^.

Inhibition of cell growth and induction of cell death are the main targets of cancer treatment. However, melanoma is one type of cancer that constantly evolves resistance to programmed cell death, which is most likely due to dysregulation of apoptosis^1^. Therefore, the induction of other forms of cell death like mitotic catastrophe, senescence and especially autophagy, is necessary and fundamental to conquer this resistance^25^,^26^. Autophagy is a dynamic cellular self-digestion process and in most cells occurs at constitutive levels to maintain internal homeostasis of cytoplasm^27^. Recent studies presented convincing proofs that autophagy defends against various diseases, for instance, cancer, aging and neurodegenerative disease^28^. Hence, induction of other death mechanisms, such as autophagy, provides a critical defensive strategy to guarantee the removal of potentially carcinogenic cells^29^. In addition, it has reported that Cur can serve as an inducer of autophagy in several cancer cells^14^,^19^,^30^. Therefore, it may be interesting to explore autophagy for melanoma treatment. In consequence, the understanding of how to tip the scales between cancer growth and death is requiring the comprehension of the intricate relationship among cell apoptosis, autophagy and other forms of cell death^31^.

Our previous observations have shown that mixed LED red and blue light phototherapy exhibited a more synergized effect than Cur alone, probably by combing the anti-proliferative and apoptosis-inducing characteristics. Hence, in the present research, we designed to investigate more deeply on the effects of such a combined dispose of human melanoma cells and try to expound the molecular mechanism of the coordinating actions. The study displayed that Cur combined with red united blue light irradiation maybe offer a potential treatment option for human cancers.

## Results

### Cur in combination with red united blue light irradiation effectively induces oxidative stress- mediated cell death in A375 cells

**Figure 1:**
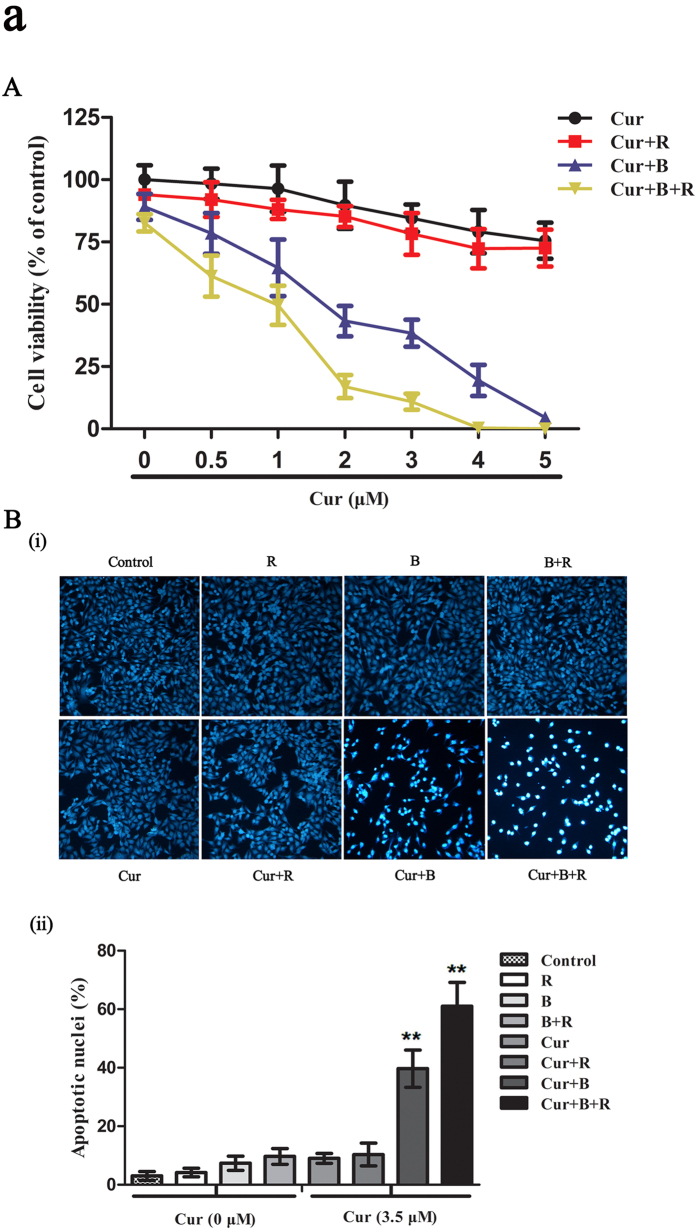
Cur in combination with red united blue light irradiation effectively induces cell death in A375 cells. (**A**) A375 cells were pre-processed with Cur (0–5 μM) for 2 h, and then irradiated with red light, blue light and combined utilization of red and blue light, or protected from light respectively. Twenty hours after the last treatment, cell viability was detected by CCK-8 assay kit. The inhibition ration is in a dosage-dependant pattern. (**B**) A375 cells were treated as described above, and then stained with bisbenzimide. (i) Nuclear changes in A375 cells treated with Cur and light irradiation; (ii) The assessment was carried out by calculating about 200 cells of each probe of light protected or irradiated cells. Bars between control groups and treated groups are remarkable different at p < 0.05(*) or p < 0.01(**) level.

First, we evaluated the A375 cell viability after treated with red united blue light irradiation in combination with Cur using CCK-8 assay. As shown in Fig. 1A, Cur alone or Cur combined with red light irradiation decreased cell viability in a dosage-dependent pattern, but the impacts were not apparent (*P* > 0.05); as well, no evident changes of cell viability were observed in cells treated with light irradiation alone (*P*>0.05); contrastively, Cur combined with blue light or red united blue light irradiation significantly enhanced the cytotoxic effects. For example, treatment with 2 μM Cur alone or combined with red light irradiation had slight effect on the cell viability. Nevertheless, treatment with 2 μM Cur combined with blue light irradiation decreased the cell viability to about 43% (*P* < 0.05); treatment with 2 μM Cur combined with red united blue light reduced the cell activity to about 20% (*P* < 0.01), causing significantly powerful inhibition of cell growth compared to Cur alone (Fig. S1). These results indicated that treatment with low concentration of Cur alone had little effect on A375 cell viability; while treatment with Cur in a combination of red united blue light irradiation caused apparent cell growth inhibition, thus could efficiently result in cytotoxicity in A375 cancer cells.

This result was also verified by microscopic examination. The images showed that A375 cells treated with Cur combined with red united blue light irradiation exhibited observable morphology changes, for instance, cells were separated from the surface due to cell shrinkage, and increased formation of apoptotic bodies, compared to those treated with Cur or light irradiation alone (Figs 1B and S2).

**Figure 2:**
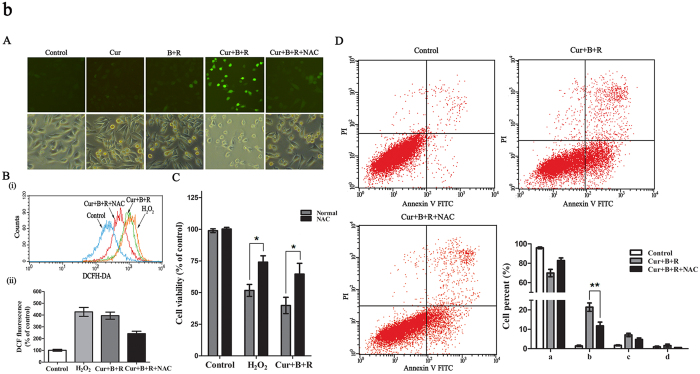
Cur in combination with red united blue light irradiation triggers cell death by means of oxidative stress. Cells were treated with Cur alone, red united blue light alone, Cur in combination of red united blue light or accessional 5 mM NAC for 20 h as described above. Then cells were incubated with 10 μM H_2_DCFDA light protected. (**A**) Cells were washed and detected by fluorescence microscope. (**B**) (i) Cells were washed and examined by flow cytometry. (ii) Mean intensity of fluorescence from DCF. (**C**) The relative proportion of cell viability. Cell viability was detected by CCK-8 assay kit. (**D**) Inhibition of oxidative stress retarded Cur combined with red united blue light irradiation induced apoptosis. Statistical analysis of apoptotic ratio was calculated via the percentage of apoptotic cells. (a, b, c and d represent normal cells, early apoptosis cells, late apoptosis cells and dead cells, respectively). Bars with different characteristic are conspicuously different at p < 0.05(*) or p < 0.01(**) level.

Because photosensitizing compounds may induce phototoxicity by generation of reactive oxygen species (ROS) and the accumulation of intracellular ROS can lead to oxidative damage of DNA, which culminates in cell cycle arrest, programmed cell death and necrotic cell death^6^,^32^, we further examined whether Cur combined with red united blue light irradiation has a similar mechanism. A375 cells were incubated with 3.5 μM Cur in combination of red united blue light irradiation in the presence of 5 mM N-acety-L-cysteine (NAC), which is a classic antioxidant, and A375 cells treated with hydrogen peroxide (H_2_O_2_) as a positive control for the ROS production. The endocellular production of oxidative stress was evaluated by means of the transformation of non-fluorescent H_2_DCF to fluorescent DCF^33^. Our results showed that there is a prominent increase of fluorescent DCF in Cur synergized red united blue light irradiation treated A375 cells (*P* < 0.05), and NAC efficiently reduced this oxidative stress (Fig. 2A,B). However, no evident changes of fluorescent signals were observed in cells treated with Cur or light irradiation alone. Meanwhile, we also inspected whether the increase of oxidative stress involves in Cur united light irradiation-induced cell death. A375 cells were treated in the presence of 5 mM NAC, as described above. As well, NAC observably retarded cell death induced by Cur combined with red united blue light irradiation (Fig. 2C). Importantly, NAC also inhibited Cur combined with red united blue light induced apoptotic cell death (Fig. 2D), suggesting that intracellular ROS function as an upstream controller to regulated apoptosis in A375 cells.

All of the above results demonstrated that Cur combined with red united blue light irradiation caused cell death due to the induction of oxidative stress. And the growth inhibitory effects of Cur and red united blue light irradiation were strongly synergized. Red united blue light irradiation enhanced the effects of Cur due to the light energy intake, making Cur under low concentrations can also exert anti-cancer efficacy.

### Cur associated with red united blue light induces reversible cell cycle arrest in A375 cells

**Figure 3:**
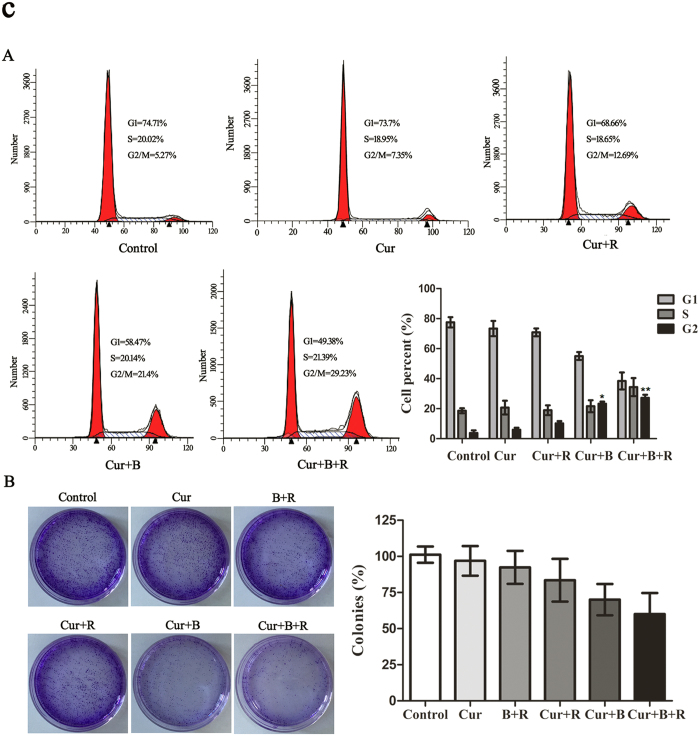
Cur associated with red united blue light induces reversible cell cycle arrest in A375 cells. (**A**) Cell cycle distribution of A375 cells treated with Cur and light irradiation. Cells were treated with Cur alone, Cur combined with red light, Cur combined with blue light and Cur synergized red united blue light irradiation, respectively. The histograms exhibit percent of cells in different phages of cell cycle. (**B**) Clonogenic survival assay of A375 cells treated with Cur in the absence or presence of light irradiation. A375 cells were treated as described above. 20 h later, the cells were collected and cultured in fresh medium at 1000 cells/well. After 9 days’ culture, cells were fixed with 4% paraformaldehyde and stained with crystal violet. Analysis of colonies per dish, represented as a percent of control. Bars between control groups and treated groups are conspicuously different at p < 0.05(*) or p < 0.01(**) level.

High concentrations of Cur have shown abilities to hinder cell cycle progression in a variety of cancer types^34^. So we inspected whether it modulates cycle kinetics in A375 cells at low concentration in combination with red united blue light irradiation. The tested results did not alter among control group, Cur alone treated group and Cur combined with red light irradiation group (Fig. 3A). Similarly, cells treated with light irradiation alone showed no obvious effects on cell cycle distribution (Fig. S3). However, Cur in combination with blue light treated groups induced G2/M cell cycle arrest, resulting in an evident increase in the G2/M phase from about 3.8% to 21.1% (*P* < 0.05). Furthermore, Cur combined with red united blue light gave rise to an even more obvious cell proliferation inhibition, where cell cycle was apparently retarded at the G2/M transition point from 3.8% to 28.2% (*P* < 0.01) (Fig. 3A).

In order to further determine whether A375 cells lose multiplication capability temporally or permanently, cell clonogenic survival assay was also carried out. As shown in Fig. 3B, when A375 cells were treated with Cur combined with red united blue light irradiation, the ability to form colonies was not obviously changed compared to the control group (*P* > 0.05), although the number of cells in each clones was reduced, indicating that Cur combined with red united blue light irradiation-arrested cells maintained the proliferation potential.

### Cur cooperates with red united blue light to induce apoptosis

**Figure 4:**
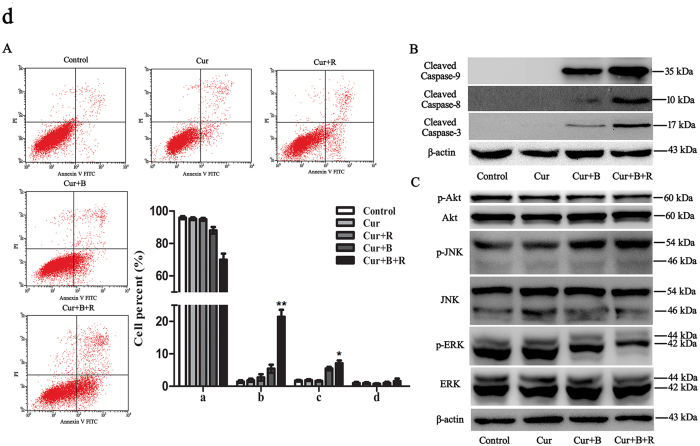
Co-induction of apoptotic cell death by Cur combined with red united blue light irradiation. (**A**) The apoptosis incidence of A375 cells were evaluated by flow cytometry. Cells were treated with Cur, Cur combined with red light, Cur combined with blue light and Cur synergized red united blue light irradiation, respectively. Statistical analysis of apoptotic ratio was calculated via the percentage of apoptotic cells. (a, b, c and d represent normal cells, early apoptotic cells, late apoptotic cells and dead cells, respectively). (**B**) Western blotting exhibition of caspases activities ability, with β-actin as a loading control. (**C**) The phosphorylation degree of JNK, ERK and Akt were analyzed by Western blotting assay, with total JNK, ERK and Akt acted as loading controls. Remarkable difference between control groups and treated groups are shown at p < 0.05(*) or p < 0.01(**).

Restraint of cell proliferation may be caused cell cycle arrest or cell apoptosis or a combined action of these pathways. So as to ascertain the possible regulatory mechanism of Cur in combination with red united blue light irradiation induced cell death, we analyzed cell apoptotic rate via flow cytometry assay. As shown in Fig. 4A, no apparent phenomenon of cell apoptosis was observed in Cur alone treated cells or Cur combined with red light treated cells. Similarly, cells treated with light irradiation alone showed tiny differences in cell apoptosis (Fig. S4). However, treatment of Cur in combination with blue light increased the apoptotic cell proportion from about 3.1% to 10.9% (*P* < 0.05). Furthermore, the combined treatment with Cur and red united blue light conspicuously increased the cell apoptotic rate from about 3.1% to 29.8%, especially cells at early stage apoptosis (*P* < 0.01) (Fig. 4A). These results displayed that Cur combined with red united blue light induced intense apoptosis in A375 cells.

Apoptosis is a course of a series of gene activation and regulation. Caspases, a family of cysteine acid proteases closely associated with apoptotic mechanisms, is an early signal of apoptosis^35^. Several studies have shown that Cur at high concentrations caused damage to different cancer cells through activating caspase pathways. In this study, we inspected both caspase-8 and caspase-9 activities via Western blotting to verify whether they were also involved. Our results showed that the treatment of Cur alone had little effect on the cleavage of caspase-8 and caspase-9 (Fig. 4B). However, the combined treatment of Cur and red united blue light obviously enhanced the activation of caspase-9 and promoted activity of caspase-8, respectively (*P* < 0.05). As caspase-3 is the final stage of apoptosis initiation shared by both pathways, we also examined its activity. The results showed that treatment of Cur alone made no difference on the cleavage of caspase-3. Whereas, combined treatment of Cur and red united blue light markedly promoted caspase-3 activation (Fig. 4B). In addition, pan-caspase inhibitor Z-VAD in some extent suppressed Cur combined with red united blue light-induced cell death (Fig. S5).

MAPK cascade activation is the center of various signaling pathways and plays a critical role in many cell proliferation related signaling pathways^36^. Therefore, we had also estimated whether the MAPK pathways are activated in Cur synergized red united blue light irradiation-treated A375 cells. The results showed that red light alone, blue light alone, red united blue light irradiation, or low concentration of Cur alone had no obvious effects on the activation of JNK and ERK (Fig. S6). Nevertheless, the combined treatment of Cur and red united blue light irradiation obviously enhanced the activation of JNK and exhibited a trivial effect on p38 (data not shown), but apparently brought down the phosphorylation level of ERK (Fig. 4C).

It is well known that Akt pathway also plays a vital role in regulating cell propagation and cell apoptosis^37^. In our study, we found that Cur alone or light irradiation alone showed no apparent efficacy on the phosphorylation level of Akt (Fig. S6). However, its phosphorylation level was significantly decreased in cells treated with Cur combined with red united blue light irradiation (Fig. 4C), indicating that Cur combined with red united blue light irradiation also induced apoptosis through inhibition of Akt pathway in A375 cells.

To sum up, all these results indicated that not only the intrinsic and extrinsic apoptosis pathways, but also the MAPKs and Akt pathways took part in the regulation of Cur combined with red united blue light irradiation-induced cell apoptosis, which included the activation of caspase-8 and caspase-9, up-regulation of phosphorylation of JNK and down-regulation of phosphorylation levels of ERK and Akt.

### Cur combined red united blue light irradiation induces autophagy in A375 cells

**Figure 5:**
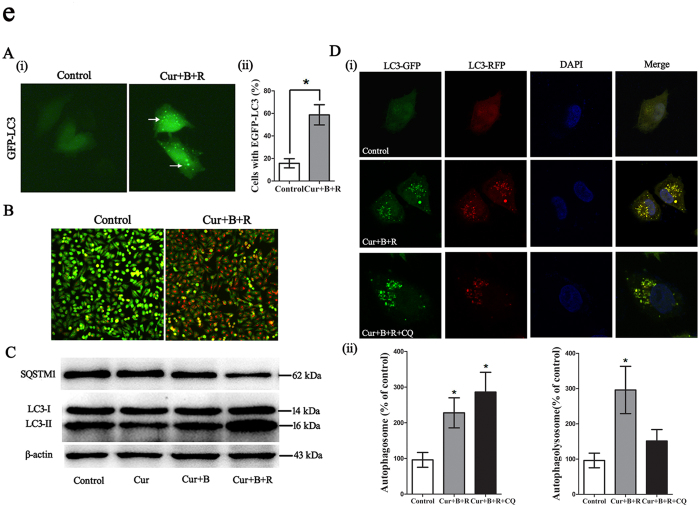
Cur combined with red united blue light irradiation induces autophagy in A375 cells. (**A**) Observation of punctuate distribution pattern of EGFP-LC3 by fluorescence Microscope. A375 cells were transfected with pEGFP-LC3 20 h before treated with Cur combined with red united blue light irradiation. (i) Cells with cytosolic green spots representing autophagsomes. (ii) The percent of cells with cytosolic EGFP-LC3 spots. (**B**) Increase of acidic vacuolar organelles (AVO) in Cur combined with red united blue light treated cells. Cells were pre-treated with Cur combined with red united blue light for 20 h, whereafter, stained with acridine orange and detected with fluorescence microscope. (**C**) Western blotting analysis of LC3 and SQSTM1. A375 cells were treated with Cur, Cur combined with blue light, Cur combined with red united blue light irradiation, respectively. The conversion of LC3-II and degradation of SQSTM1 were tested by immunoblotting, with β-actin as a loading control. (**D**) Detection of autophagic flux. Representative confocal fluorescent images of cells transfected with TagRFP-EGFP-LC3 followed by the treatment with Cur combined with red united blue light irradiation for 20 h. CQ treated cells used as a positive control. The nuclei were stained with DAPI. (i) Puncta of autophagosome (green fluorescence) and autophagolysosme (red fluorescence) were monitored using confocal microscope. (ii) Statistic results of autophagosome or autophagolysosome in control, Cur combined with red united blue light irradiation or both Cur combined with red united blue light irradiation and CQ treated cells. Data are represented as a percent of control. Bars between control groups and treated groups are conspicuously different at p < 0.05(*) or p < 0.01(**) level.

Considering that Cur can also act as an autophagy elicitor^30^,^38^, we next detected whether Cur combined with red united blue light irradiation induces autophagy in A375 cells. The punctuated distribution of GFP-LC3 is a well-accepted marker of autophagy. Here we revealed that treatment of A375 cell with Cur at a low concentration of 3.5 μM, combined with red united blue light irradiation enhanced the per centum of cells with EGFP-LC3 particles (Fig. 5A). Besides, we also detected the autophagosomes through acridine orange (AO) staining, which were significantly enhanced after the treatment of Cur combined with red united blue light irradiation (Fig. 5B).

In addition, we analyzed the expression level of LC3 and SQSTM1, which are reliable markers of autophagosome^39^,^40^,^41^. The quantity of LC3-II increases during autophagosome come into being, while decreases in the course of autophagosome-lysosome fusion. The conversion of LC3-I to LC3-II is a sign of autophagic activity^39^. We tested the combined effect of Cur pretreatment and red united blue light irradiation on LC3 and SQSTM1 conversion in A375 cells. The results showed that treatment of A375 cells with Cur at a low concentration combined with blue light irradiation slightly increased the conversion of LC3-I to LC3-II; and this effect was obviously further enhanced when cells were treated with Cur combined with red united blue light irradiation compared with the control cells (Fig. 5C). Moreover, SQSTM1 was decreased by the treatment of Cur combined with blue light and red united blue light irradiation, respectively.

Autophagy markers accumulation may represent either autophagy induction or, alternatively, impaired clearance of autophagosome^39^,^42^. To further determine the effect of Cur combined with red united blue light irradiation on autophagy, a tandem fluorescent-tagged LC3 (mRFP-EGFP-LC3), which was sensitive in detecting the accumulation of autophagosome and autophagolysosome based on different pH stability of EGFP and mRFP fluorescent proteins, was expressed in A375 cells to monitor autophagic flux. After cell treatment, both autophagosomes and autophagolysosomes were markedly augmented, indicating an increased autophagic flux in Cur combined with red united blue light irradiation treated cells. While, cells combined treatment with autophagy inhibitor CQ triggered a conspicuous increase of autophagosome but no significant change in the formation of autophagolysosome (Fig. 5D).

Taken together, all these data demonstrated that Cur combined with red united blue light irradiation induced autophagy in A375 cells.

### Inhibition of autophagy enhances Cur combined with red united blue light irradiation-induced cell death

**Figure 6:**
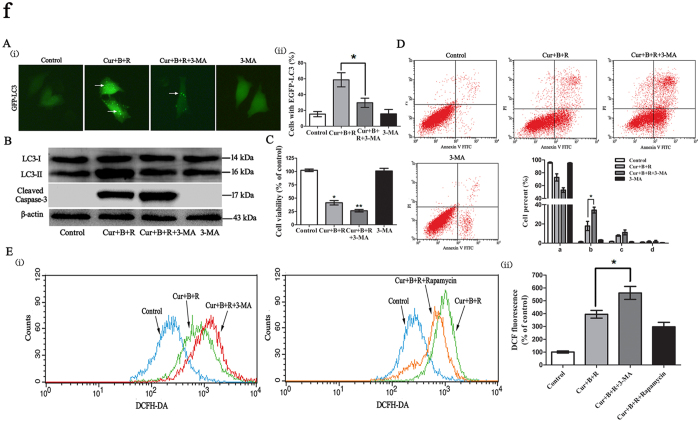
Inhibition of autophagy enhances Cur combined with red united blue light irradiation induced cell death. (**A**) Observation of punctuate pattern of EGFP-LC3 by fluorescence Microscope. A375 cells were transfected with pEGFP-LC3 20 h before treated with Cur combined with red united blue light irradiation or simultaneously treated with both Cur combined with red united blue light irradiation and 3-MA. (i) Cells with cytosolic green spots representing autophagsomes. (ii) The percent of cells with cytosolic EGFP-LC3 spots. (**B**) Analysis of LC3II/I conversion and caspase 3 activation in Cur combined with red united blue light treated cells. A375 cells were treated with Cur combined with red united blue light irradiation, simultaneously treated with both Cur combined with red united blue light irradiation and 3-MA, or 3-MA alone, respectively. The conversion of LC3-II and activation of caspase-3 were tested by immunoblotting, with β-actin as a loading control. (**C**) The impact of the combination of Cur combined with red united blue light and 3-MA on cell viability. (**D**) Inhibition of autophagy enhances apoptotic cell death in A375cells. Statistical analysis of apoptotic ratio was calculated via the percentage of apoptotic cells. (a, b, c and d represent normal cells, early apoptotic cells, late apoptotic cells and dead cells, respectively). (**E**) Influence of autophagy on Cur combined with red united blue light induced oxidative stress. (i) Cells treated with Cur combined with red united blue light and 3-MA or Rapamycin. (ii) Quantitative analysis of ROS production detected via flow cytometry. Significant difference between groups are shown at p < 0.05(*) or p < 0.01(**).

Many reports have shown that anticancer compound-induced autophagy can either protect cancer cells against death or give rise to cancer cell death^43^,^44^. Therefore, we investigated the impact of Cur combined with red united blue light irradiation-induced autophagy in cell death. First, we inspected the effect of 3-methyladenine (3-MA), an inhibitor of phosphatidylinositol 3-kinase class III (PI3k class III)^45^, on the inhibition of autophagosome formation. The results showed that 3-MA decreased the amount of cells containing EGFP-LC3 in the group treated with Cur combined with red united blue light (Fig. 6A). Untreated pEGFP-LC3 transfected cells under normal light were shown in Fig. S7.

Identically, Western blotting results also revealed that LC3 conversion was declined in cells treated with both Cur combined with red united blue light irradiation and 3-MA (Fig. 6B). However, 3-MA alone had little impact on LC3 conversion. Subsequently, we investigated the effect of autophagy in Cur combined with red united blue light-induced cell death. The results showed that when Cur combined with red united blue light irradiation induced autophagy was inhibited by 3-MA, an obvious increase of cell death appeared (Fig. 6C). Additionally, when cells were disposed with both Cur combined with red united blue light irradiation and 3-MA, apoptotic cells were markedly enhanced (Fig. 6D), accompanied with a strong increase of caspase-3 activation (Fig. 6B). These results exhibited that inhibition of autophagy increased Cur combination with red united blue light irradiation-induced apoptotic cell death.

In the meantime, we were curious about whether autophagy has an effect on Cur combined with red united blue light irradiation generated cellular ROS. The results showed that cellular ROS was enhanced in the A375 cells treated with both Cur combined with red united blue light irradiation and autophagy inhibitor 3-MA, compared to that of cells treated with Cur combined with red united blue light irradiation alone (*P* < 0.05) (Fig. 6E). Simultaneously, cellular ROS was reduced in the A375 cells treated with both Cur combined with red united blue light irradiation and autophagy inductor rapamycin, although the difference was not significant. These results indicated that autophagy were capable of reducing Cur combined with red united blue light irradiation-evoked cell death by decreasing cellular ROS.

### Inhibition of autophagy converts Cur combined with red united blue light irradiation-induced reversible cell cycle arrest to senescence in A375 cells

**Figure 7:**
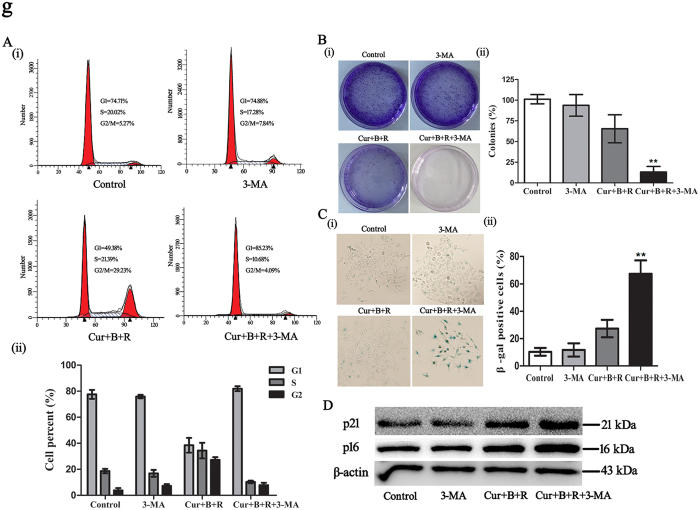
Inhibition of autophagy converts Cur combined with red united blue light irradiation-induced reversible cell cycle arrest to senescence in A375 cells. (**A**) Inhibition of autophagy retarded Cur combined with red united blue light irradiation induced cell cycle arrest. (i) Cell cycle distribution of A375 cells that treated with 3-MA alone, Cur combined with red united blue light or simultaneously treated with both Cur combined with red united blue light irradiation and 3-MA, respectively. (ii) Quantification of cell cycle analysis. (**B**) Colony formation assay of A375 cells with treatment of Cur combined with red united blue light and 3-MA. (i) Representative graphics of clonogenic survival assay. (ii) Analysis of colonies per dish, represented as a percent of control. (**C**) Analysis of senescent cell percent upon treatment of Cur combined with red united blue light and 3-MA. (i) Representative graphs of cells stained for β-gal activity. (ii) Quantification of the percent of SA-β-gal positive cells. Bars between control groups and treated groups are remarkable different at p < 0.05(*) or p < 0.01(**) level. (**D**) Western blotting analyses of senescent markers p21 and p16, with β-actin as a loading control.

To further investigate the impact of autophagy in Cur combined with red united blue light irradiation-induced reversible cell cycle arrest, we performed cell treatment with both Cur combined with red united blue light irradiation and 3-MA analysis as described previously. The results showed that inhibition of autophagy obstructed Cur combined with red united blue light irradiation-induced cell cycle arrest. As shown in Fig. 7A, cells treated with both Cur combined with red united blue light irradiation and 3-MA exhibited an evident decline in the G2/M phase cell cycle arrest. In addition, we also surveyed the invertibility of cells treated with Cur combined with red united blue light irradiation and 3-MA, by colony formation experiment. As shown in Fig. 7B, cells treated with Cur combined with red united blue light irradiation or 3-MA alone recovered proliferation. Nevertheless, cells simultaneously treated with Cur combined with red united blue light irradiation and 3-MA was unable to propagate and develop into colonies even removing drugs away. Interestingly, cells treated with both Cur combined with red united blue light irradiation and 3-MA indeed revealed an aging morphology, which was testified by β-gal staining (Fig. 7C). Next, we also investigated changes of senescent markers p21 and p16 in Cur combined with red united blue light irradiation and 3-MA treated A375 cells. The results showed that both p16 and p21 were significantly up-regulated (Fig. 7D).

Hence, inhibition of autophagy led Cur combined with red united blue light irradiation-induced reversible proliferation arrest to lose the potential for proliferation and give rise to senesce in A375 cells.

## Discussion

Curcumin, exhibits pleiotropic effects, such as anti-oxidant, anti-carcinogenic, anti-inflammatory, apoptosis-accelerating and radiosensitive attributes^14^,^46^,^47^. It has been proved to be riskless, tolerated and exerts no adverse side reactions even though at a high density^46^,^48^. Studies have shown that Cur exerts its cytotoxic effect through regulation of multiple pathways and induction of diverse modulations of cell proliferation arrest and cell death, and the pleiotropic activities are greatly inconstant on account of cell type, dosage, timing and mode of action^45^,^49^. For all that, the majority of these studies employed Cur in the middle or high concentration range, on account of that the application is excessively restricted due to its poor bioavailability concernes with its low assimilation and rapid metabolism^50^. In our previous studies, we have shown that Cur in combined with red united blue light irradiation, even at the low concentration, modulates cell apoptosis and proliferation in skin keratinocytes^51^. Our research indicated that red light united blue light irradiation, combined the apoptosis-accelerating and anti-proliferative abilities to strengthen the irritation of the target photosensitizer and to arrive the photodynamic target sites.

In this study, we demonstrated that Cur synergized with red united blue light irradiation efficiently induced ROS-mediated cell death. However, there were no evident changes of ROS level in cells treated with Cur or light irradiation alone (data not shown). When cells were treated with Cur combined with red united blue light irradiation in the presence of 5 mM NAC, the oxidative stress was efficiently reduced, coupled with significantly decreased Cur combined with red united blue light irradiation-induced cell death. All these results suggested that Cur combined with light irradiation-promoted cell death is induced by oxidative stress. ROS has been perceived as a crucial regulatory factor of apoptosis by dint of adjusting and controlling multiple signaling pathways, including both intrinsic and extrinsic apoptosis pathways^52^,^53^. Moreover, it has also been reported that ROS gives rise to down-regulation of the Akt and ERK pathways^54^. Our results showed that the combined treatment of Cur and red united blue light irradiation gave rise to apoptosis in A375 cells, demonstrated by the formation of apoptotic bodies, the activation of caspase-8 and caspase-9 and the regulation of phosphorylated JNK, ERK and Akt, without disrupting the cell membrane integrity.

Unlike cells treated with Cur alone or single light irradiation, Cur synergized with red united blue light irradiation not only remarkably enhanced the activation of caspase-9, but also promoted the activity of caspase-8. Caspase-9 is a principal originator of the intrinsic pathway, while, caspase-8 is a dominating initiator protease gathered to the death-inducing signal complex in the extrinsic pathway, both are the important pathways involved in cell apoptosis^55^. Caspase activation is an inchoate signal of apoptosis, which plays a vital role in the modulation of apoptosis^35^. Synchronously, these results were deeper confirmed by flow cytometric results. The combined treatment with Cur and red united blue light irradiation observably increased cell apoptotic rate, which may sensitize cells to apoptosis by activating caspase pathways. From the above, our present studies indicated that both extrinsic and intrinsic mediated apoptotic pathways took part in Cur combined with red united blue light irradiation-induced cell apoptosis.

Many studies have shown that MAPK signaling pathway exerts a major role in the regulation and control of cell progression and apoptotic cell death^36^. JNK, ERK and p38 pathways are the three most important pathways in MAPK signaling channel. JNK and p38 could stimulate apoptosis; nevertheless, ERK, extracellular regulated protein kinases, was able to obstruct cell apoptosis via preventing caspases excitation^37^. Our results showed that the combined treatment of Cur and red united blue light irradiation obviously up-regulated the phosphorylation level of JNK and exhibited a trivial effect on p38; while apparently brought down the phosphorylation level of ERK. Furthermore, the Akt signaling pathway plays a vital role in cell proliferation and cell apoptosis, as well^56^. Some studies have shown that Akt could retard the expression of pro-apoptotic proteins to change the cell viabilities. In our studies, we found that the phosphorylation level of Akt was significantly decreased when cells were treated with Cur combined with red united blue light irradiation.

Taken together, consistent with previous research, our results indicated that the up-regulation of phosphorylated JNK played a pivotal role in facilitating apoptosis; however, the reduced phosphorylation level of ERK and Akt may in favor of promoting apoptosis.

The last but not least, Cur combined with red united blue light irradiation also promoted autophagy in A375 cells. Autophagy, a dynamic catabolism process that transports cytoplasmic substance to lysosomes by virtue of autophagosomes, is involved in many pathological and physiological procedures^57^. Autophagy can either accelerate cell death or protect cancer cells from death, which has become a key factor for cancer treatment^58^. Our results showed that, Cur combined with red united blue light irradiation-induced autophagy has an adverse effect on apoptosis, since inhibiting of autophagy enhanced cell apoptosis. Besides, Cur combined with red united blue light irradiation-induced autophagy also played a part in the regulation of cellular ROS, by reducing cellular ROS to decrease cell death.

Uncontrolled cell proliferation is a conspicuous hallmark of cancer cell. Therefore, triggering proliferation arrest of cancer cell is a crucial means for cancer cell regulation^43^. Inhibition of cancer cell proliferation may lead to reversible cell cycle arrest or irreversible cell proliferation arrest (senescence)^59^. Because reversible proliferation-arrested cancer cells may also have the potential to recurrence and metastasis, it is hazardous for cancer therapy^60^. However, senescence in cancer cells might be an obstacle to cancer cell proliferation^59^.

Here, our research showed that Cur, at the relative low concentration of 3.5 μM, synergized with red united blue light irradiation retarded A375 cell proliferation through a process that involved in autophagy. Cur combined with red united blue light irradiation-induced autophagy played an important role in the G2/M cell cycle arrest, because the suppression of autophagy significantly counteracted this impact. Furthermore, inhibition of autophagy turned Cur combined with red united blue light irradiation-induced reversible cell cycle arrest to lose the potency for proliferation and triggering irreversible cell arrest in A375 cells, which hints that autophagy exerts a protective effect on Cur combined with red united blue light irradiation-induced cell death. Whereas the molecular harmonizing mechanisms among apoptosis, autophagy and cell cycle regulation are incompletely understood, cell apoptosis and autophagic cell death may exist in a synergetic or opponent relationship depending on the cellular circumstance. Therefore, the mutual effect among apoptosis, autophagy and cell cycle regulation deserved more and more attention.

In summary, we supposed a signal regulating network for the synergistic effects of Cur and red united blue light irradiation. Cur pretreatment combined with red united blue light irradiation strengths intracellular level of ROS. After that, ROS serves as an upstream regulatory factor to regulate the phosphorylation level of JNK, ERK and Akt^53^,^54^, and then they trigger cell apoptosis by regulating the activation of caspase-8 and caspase-9 pathways. Furthermore, excessive production of ROS was able to prevent the activation of Akt due to negative feedback regulation, which may facilitate cell apoptosis. In addition, Cur synergized with red united blue light irradiation also induced autophagy, and the pharmachological suppression of autophagy not only enhanced the cellular ROS, but also turned reversible arrested cells to senescence-like proliferation arrest in A375 cells, thus decreasing the possibility that injured cells could avoid apoptosis and actuate vicious conversion.

In a word, our present consequences demonstrated that the combined employment of red united blue light irradiation enhanced the cellular assimilation of Cur, by integrating the apoptosis-inducing and anti-proliferative properties to effectively compensate the low bioavailability of Cur. Cur combined with red united blue light induced autophagy was indispensable for sustaining proliferation capability and inhibition of autophagy transformed reversible cell cycle arrest to senescence-like cycle arrest. Because senescence gives rise to a general obstruction to tumor genesis, induction of apoptosis and other cell death approaches, such as autophagy or in-reversible cell cycle arrest, is on behalf of a valid defense tactics to guarantee the elimination of damaged and potentially cancerogenic cells.

## Materials and Methods

### LED source

The output curve of LED light source was detected by the Chinese National Institute of Metrology (Fig. S8). Irradiation dosages were: blue light-emitting diode lighting with a maximum strength at 405 nm (161 μW/cm^2^nm), 10 min of irradiation, accumulated dose 1.604 J/cm^2^; red light-emitting diode illumination with a top intensity at 630 nm (300 μW/cm^2^nm), 10 min of irradiation, accumulated dose 3.409 J/cm^2^.

### Reagents and antibodies

Antibodies against cleaved caspase-3, cleaved caspase-8, cleaved caspase-9, AKT, phospho-Akt (p-Akt), ERK, phospho-ERK (p-ERK), JNK, phosphor-JNK (p-JNK) (Cell Signaling Technology, Boston, MA, USA); anti-p62/SQSTM1(MBL); antibody against LC3B, Curcumin, 3-methyladeine (3-MA), chloroquine diphosphate (CQ), Rapacymin, the fluorescent dye Acridine Orange (AO) and Crystal violet (Sigma-Aldrich, St. Louis, MO, USA); Lipofectamine 3000 (Invitrogen, Carlsbad, CA, USA); pre-stained protein standards (Fermentas, Lithuania); Human Annexin V Apoptosis Detection Kit and cell cycle test kit (BD Biosciences, San Jose, CA); β-actin antibody (Santa Cruz Biotechnology, USA); Senescence β-Galactosidase Staining Kit (Beyotime Biotechnology). Cur was dissolved with dimethyl sulfoxide (DMSO) and the storage concentration was 20 mM/ml; Rapacymin was solved in DMSO; 3-MA, CQ and AO were solved in ultrapure water.

### Cell culture and irradiation

In this study, melanoma cancer cell line A375 was used. A375 cells were acquired from The Cell Bank of Chinese Academy of Science (Shanghai). Cells were trained in DMEM (Dulbecco’s modified eagle medium) with 1% P/S solution (Gibco, Karlsruhe, Germany) and 10% FBS. All cells were cultivated in a humidified incubator with 37 °C and 5% CO_2_.

A375 melanoma cells were pre-processed with Cur for 2 h and then irradiated with LED array at an altitude about 25 mm. Before irradiation, PBS was substituted for the medium to avert the production of photochemical compounds. All controls were stored in PBS and kept light-protected to ensure the identical experimental conditions. Further, cells were cultured for the indicated times. In order to investigate whether autophagy was induced in Cur synergize red united blue light irradiation treated A375 cells, we also treated A375 cells with pharmacological autophagy inhibitor (3-MA, CQ) or autophagy inducer (Rapamycin). 3-MA (2 mM), CQ (10 μM) or Rapa (1 μM) was added 2 h before the treatments with Cur.

### Cell proliferation

Cell proliferation was tested by CCK-8 assay according to previously introduced^10^. A375 cells were cultivated in 96-well plates at a cell quantity of 6 × 10^3^ cells/well twenty hours before pre-processed with 2 h of Cur (0–10 μM). After pre-procession, A375 cells were irradiated with red light, blue light or red united blue light as introduced above. After treatment, cell viability was measured with adding 10 μL/well CCK-8 solution (5 mg/ml in medium) and incubated for 2 h. The quantity of formazan dye catalyzed by dehydrogenases is directly comparable to the amount of viable cells. Ultimately, the color density of formazan was inspected at 450 nm with a micro-plate reader (Spectra Max 190; Molecular Devices, Sunnyvale, CA).

### Measure of ROS generation

The relative level of intracellular ROS was assessed by means of the probe 2′, 7′-dichlorofluorescin-diacetate (H2DCF-DA, Sigma). Briefly, 2 × 10^5^ cells were seeded in plates of 35 mm 20 h before treated with Cur and light irradiation as described above. After treatment, cells were collected and then washed with PBS and re-suspended in PBS that containing 10 μM of H2DCF-DA for 15 min light protected. After that, cells were washed three times with PBS and immediately measured by flow cytometric analysis (Becton Dickinson, San Jose, CA, USA). Relative fluorescence intensity of treated cells was indicated as percent of control.

### Flow cytometric analysis

To survey the effectiveness of Cur in combination with red united blue light irradiation on A375 melanoma cells, we evaluated the cell cycle distribution and apoptotic proportion by flow cytometric analysis. The distribution of cell cycle was monitored by specialized detection kit for cell cycle (Becton Dickinson, San Jose, CA, USA). About 3 × 10^5^ cells were seeded in plates (35 mm) 20 h before treated with Cur and light irradiation as described above. Before processing, cells have been synchronized in the same cycle/G0 phase by means of starving in the DMEM medium with 0.5% serum overnight. Cells were harvested after treatment and washed by PBS, incubated in 10 min of solution A (trypsin buffer) in the dark at room temperature, then sequentially added solution B (RNase and trypsin inhibitor buffer) and solution C (propidium iodide dye solution). Flow cytometer was used for the detection of DNA content. The percentage of cells in G0/G1, S, G2/M phases was described as column diagrams.

Apoptosis occurrence was inspected by Annexin V/PI double-staining kit. For apoptosis analysis, cells were seeded at 3 × 10^5^ cells per well in 35 mm plates, followed by treatment as described above. Cells were harvested after treatment and washed with PBS, then re-suspended with binding buffer. After this, firstly cells were incubated with Annexin V solution light protected and 15 min after incubation propidium iodide (PI) dye was added just before analyzing by flow cytometer. Apoptotic cells, including those early and late apoptotic cells, were counted and regarded as a proportion of the whole cell abundance.

All of the experimental results were conducted three times and analyzed by means of Cell Quest software (Becton Dickinson, CA, USA). 1.2 × 10^4^ cells were collected for each experiment.

### Transient transfection and fluorescence microscopy

Microtubule-associated protein 1 light chain 3 (LC3), is associated to the autophagosome membranes after processing^40^. About 2 × 10^5^ cells were seeded in 6 well plates 20 h before transfection. A375 cells were transfected with lipofectamine 3000 in accordance with the manufacturer’s instruction. The aggregation and dispersion of fluorescent LC3 spots were detected using a fluorescence Microscope (Zeiss, Germany). Transfected cells were managed as introduced above, then the number of LC3 puncta was calculated no less than 100 green cells per well.

### Monitoring autophagic flux

The formation of autophagosome and autophagy flux in Cur combined with red united blue light irradiation treated A375 cells were monitored using plasmid encoding TagRFP- EGFP -LC3. Autophagic flux is increased when both yellow and red puncta are augmented in cells. The A375 cells were grown on coverslips and transfected with Lipofectamine 3000 according to the manufacturer’s instruction. Then cells were treated with either Cur combined with red united blue light irradiation or both Cur combined with red united blue light irradiation and CQ for 20 h. Then nuclei were stained with DAPI. The distribution and accumulation of fluorescent images were recorded using Laser Scanning confocal microscopy (Leica, Germany).

### Acridine orange staining

The formation of acidic vesicular organelles (AVOs) is a representative character of autophagy and its development manifests the maturation of autophagosomes^57^. For the sake of examining whether autophagy was participated in the process of Cur synergized red united blue light irradiation-induced cell death, we inspected AVOs in the cytoplasm by acridine orange (AO), which is closely associated with autophagy. About 2 × 10^5^ cells were plated in each well of a 6-well plate, followed by treatment with 3-MA and Cur in combination with red united blue light irradiation as described above. Afterwards cells were incubated with AO for 20 min light protected at room temperature, then visualized with a fluorescence microscope (Zeiss, Germany).

### Clonogenic survival assay

To make sure whether A375 cells lost multiplication potential permanently or temporarily, clonogenic survival assay was performed 20 h after treatment as described above. Cells were washed and collected by trypsinization. Then one thousand cells were seeded in 35 mm plate and incubated in fresh medium for an extra 9 days. Cells were stained with 0.05% crystal violet. The numbers of colonies were calculated and used to illuminate the possible long term effects on A375 cells.

### β-galactosidase Staining

The SA β-gal activity was detected by Senescence β-galactosidase staining kit according to the manufacture’s introduction. X-Gal was used as a substrate, and the senescence associated β-galactosidase activity increased with aging cells. Positive cells were stained aquamarine green. Results are presented as proportion of SA β-gal positive cells to total cells.

### Western blot

Total proteins were scraped into RIPA lysis buffer with protease inhibitors and sonicated on ice, then measured protein concentration by the Bradford Assay kit (Bio-Rad). After electrophoresis, proteins (20 μg/lane) were transferred to PVDF membrane (Millipore, Bedford, MA). After this, the membranes were blocked with 5% non-fat milk (dissolved in TBST buffer) for 1 h at room temperature and then incubated with specific antibodies at 4 °C overnight. After washed with TBST for three times, the membranes were incubated in corresponding secondary antibodies at room temperature for 1 h. Ultimately, protein bands were visualized by chemiluminescent ECL assay kit and the consequences were analyzed via the specialized software.

### Statistical analysis

The statistical significance between experimental values were determined by either Student’s *t*-test or the one-way ANOVA analysis in GraphPad prism 5. The level of *P* < 0.05(*) or *P* < 0.01(**) was perceived statistically significant.

## Additional Information

**How to cite this article**: Niu, T. *et al*. Inhibition of Autophagy Enhances Curcumin United light irradiation-induced Oxidative Stress and Tumor Growth Suppression in Human Melanoma Cells. *Sci. Rep.* **6**, 31383; doi: 10.1038/srep31383 (2016).

## Supplementary Material

Supplementary Information
