# Supplementary material for: Inhibition of Autophagy Enhances Curcumin United light irradiation-induced Oxidative Stress and Tumor Growth Suppression in Human Melanoma Cells
Source: Sci Rep. 2016 Aug 9;6:31383. doi: 10.1038/srep31383 (PMC4977547; doi:10.1038/srep31383)

**Inhibition of Autophagy Enhances Curcumin United light irradiation-induced Oxidative Stress and Tumor** **Growth Suppression in Human Melanoma Cells**

Tianhui Niu^1^*, Yan Tian^2^, Zhusong Mei^1^, Guangjin Guo^1^.

1 Aviation Medicine Research Laboratory, The General Hospital of the Air Force, Beijing, China

2 Department of Dermatology, The General Hospital of the Air Force, Beijing, China

* Corresponding author

E-mail: [niuhui81@126.com](mailto:tianyan311@126.com)

**Figure S1 Synergistic effects of Cur combined with red united blue light irradiation on cell viability of A375 cells.**

A375 cells were pre-processed with Cur (2 μM) for 2 h, and then irradiated with red light, blue light and combined utilization of red and blue light, or treated with light irradiation alone respectively. Twenty hours after the last treatment, cell viability was detected by CCK-8 assay kit.

**
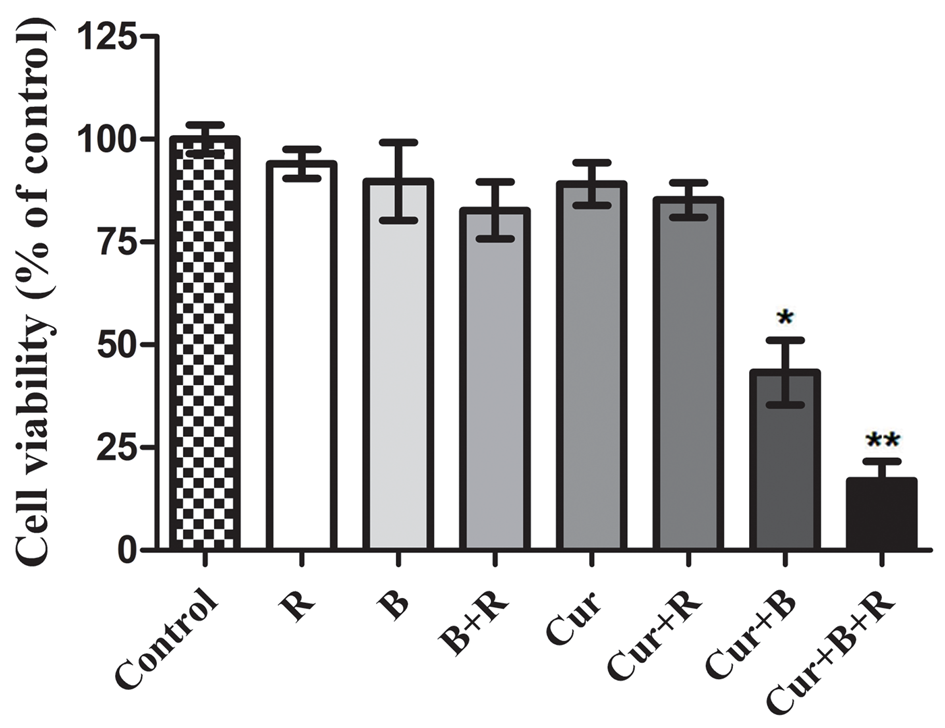
**

**Figure S2 Synergistic effects of Cur combined with red united blue light irradiation on cell morphology.**

Morphological changes of cells treated with red light alone, blue light alone, red united blue light irradiation alone, Cur, Cur combined with red light, Cur combined with blue light, Cur synergized with red united blue light respectively, as examined by fluorescence microscope.


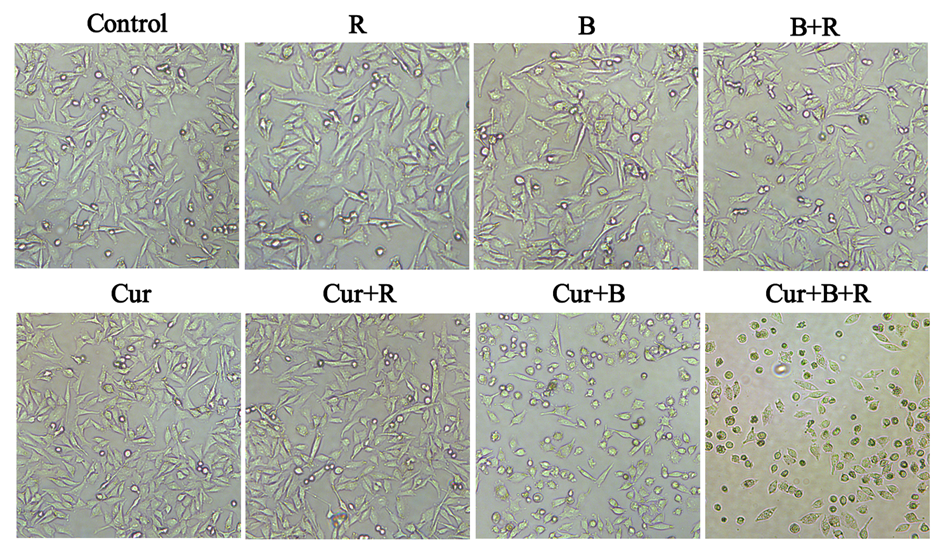


**Figure S3 Effect of light irradiation alone on cell cycle distribution.**

Cells were treated with red light alone, blue light alone and red united blue light irradiation, respectively. The histograms exhibit percent of cells in different phages of cell cycle.

**
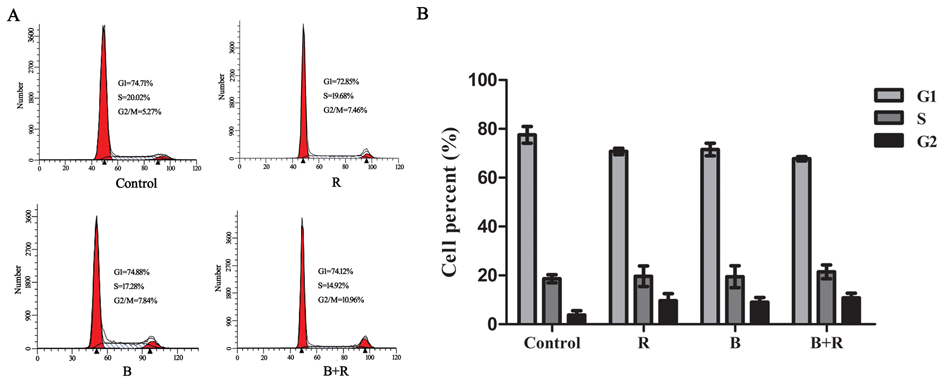
**

**Figure S4 Effect of light irradiation alone on apoptotic cell death.**

The apoptosis incidence of A375 cells were evaluated by flow cytometry. Cells were treated with red light alone, blue light alone and red united blue light irradiation, respectively. Statistical analysis of apoptotic ratio was calculated via the percentage of apoptotic cells. (a, b, c and d represent normal cells, early apoptotic cells, late apoptotic cells and dead cells, respectively).


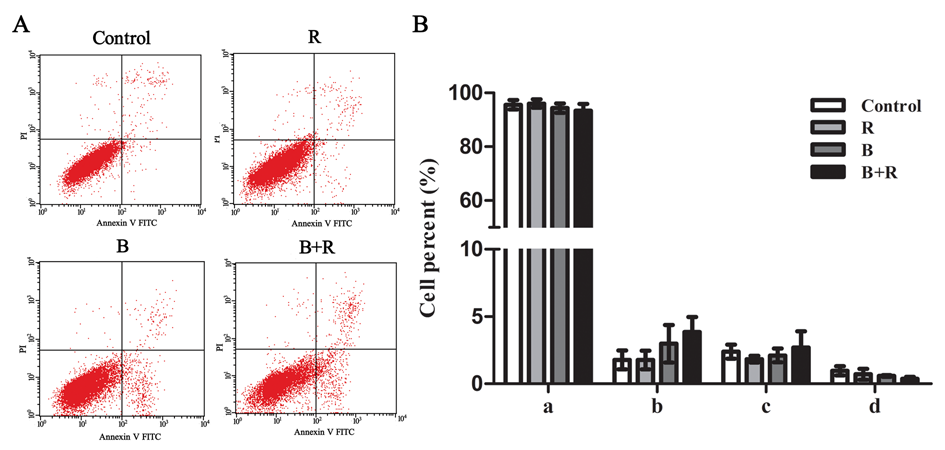


**Figure S5 Effect of caspase inhibitor Z-VAD on Cur combined with red united blue light irradiation-induced apoptotic cell death.**

Cells were treated with Cur combined with red united blue light irradiation, in the presence of caspase inhibitor Z-VAD or not. Caspase inhibitor Z-VAD suppressed Cur combined with red united blue light irradiation induced cell apoptosis, to a certain extend.


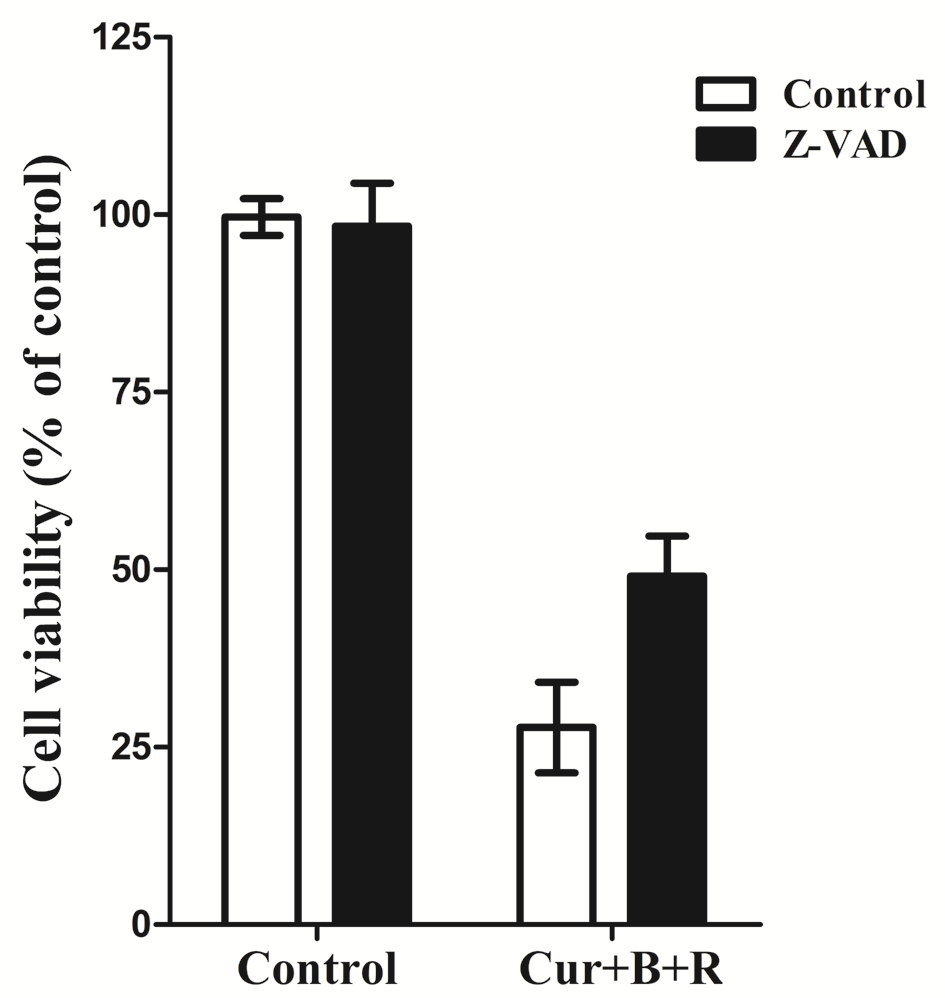


**Figure S6 Effect of light irradiation or Cur alone on the phosphorylation degree of JNK, ERK and Akt.**

Cells were treated with red light alone, blue light alone and red united blue light irradiation, respectively. The phosphorylation degree of JNK, ERK and Akt were analyzed by Western blotting assay, with total JNK, ERK and Akt acted as loading controls.

**
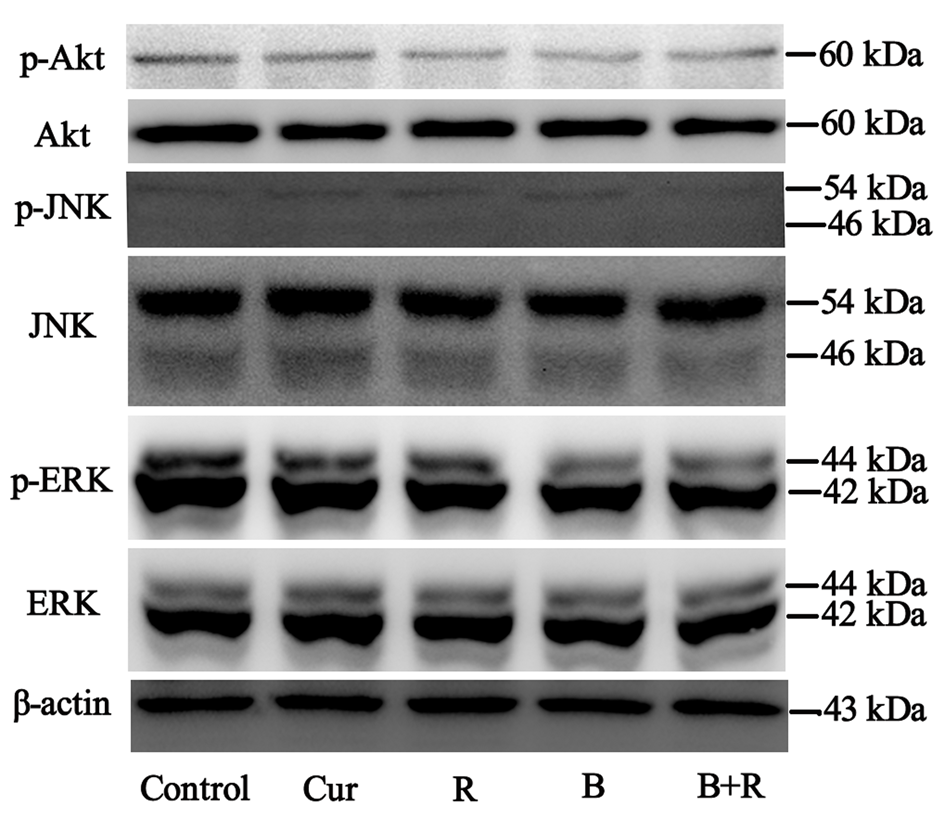
**

**Figure S7 Representative image of cell transfected with pEGFP-LC3 under normal light.**

A375 cells were transfected with pEGFP-LC3 for 20 h. Then detected by fluorescence Microscope under normal light, before treated with Cur combined with red united blue light irradiation or simultaneously treated with both Cur combined with red united blue light irradiation and 3-MA.

**
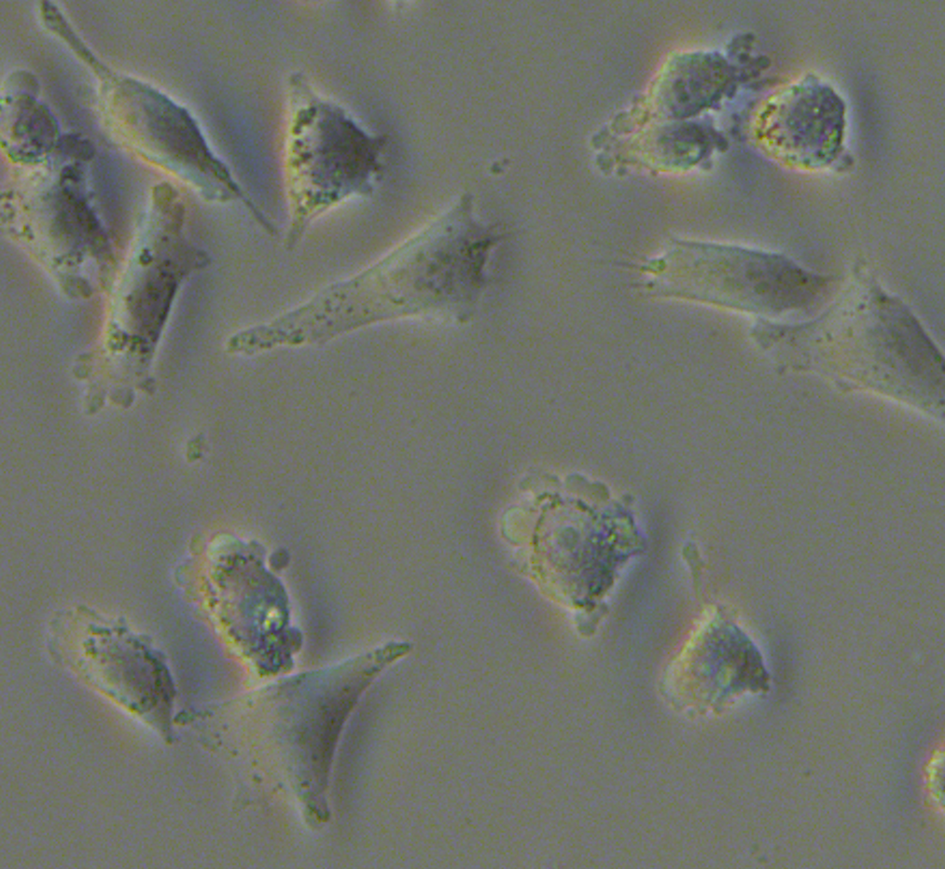
**

**Figure S8 Inspection map of LED light source.**

1. The peak of blue light is at 405 nm; (B) The maximum wave length of red light is at 630 nm.


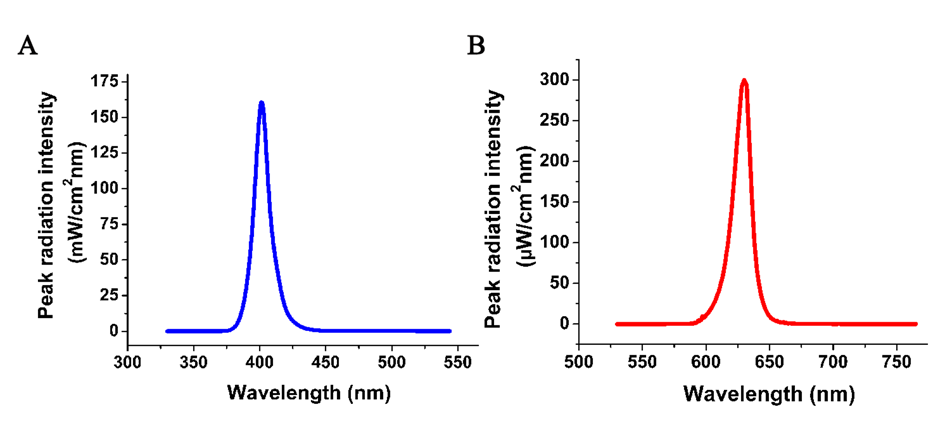


**Figure S9 Densitometry analysis of pAkt.**

Cells were treated with Cur, Cur combined with blue light and Cur synergized red united blue light irradiation, respectively. Bars with different characters are statistically different at p<0.05(*) or p<0.01(**) level.


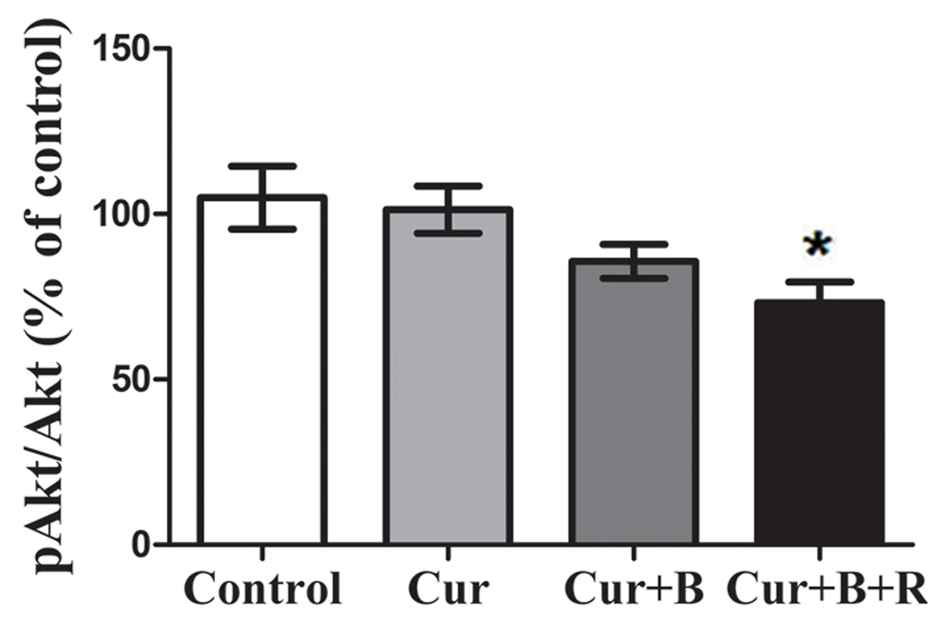

Supplement: Supplementary Information [file srep31383-s1.doc]
